# Genomic Profiling of Antibody Repertoire in Rongchang Pigs Uncovers Conserved V(D)J Gene Usage and Breed-Specific Diversification

**DOI:** 10.3390/genes17030262

**Published:** 2026-02-26

**Authors:** Qiao Li, Meng Wu, Xueqin Liu, Xingping Wu, Chuanxiang Ding, Liangpeng Ge, Hailin Zhang, Jing Sun

**Affiliations:** 1 National Center of Technology Innovation for Pigs (Chongqing), Chongqing 402460, China; 19111279846@163.com (Q.L.); wumengheling@163.com (M.W.);; 2 Institute of Bioengineering, Chongqing Academy of Animal Sciences, Chongqing 402460, China

**Keywords:** Rongchang pigs, antibody repertoire, *IGHV*/*IGHD*/*IGHJ* genes usage, HCDR3 diversity

## Abstract

Background: Pigs are economically critical livestock andan optimal model for investigating the development and diversification of antibody repertoires. The Rongchang (RC) pig, a nationally protected indigenous breed in China, possesses unique genetic characteristics, yet genomic-level research on its antibody repertoire remains limited, as most porcine antibody studies have focused on Landrace pigs. Methods: To decipher the genetic features of the antibody library in RC pigs, we used immunogenomic high-throughput sequencing to systematically analyze the antibody repertoires of five healthy purebred pig breeds, including two indigenous breeds (RC and BM) and three commercial breeds (Yorkshire, Duroc, and Landrace), with a focus on comparing conserved patterns and breed-specific differences in V(D)J gene utilization between Rongchang pigs and the other four breeds. Results: All five breeds exhibited a strong preference for a conserved subset of core *IGHV* genes. Notably, this study detected functional *IGHJ3* expression (0.40.8%) in all examined breeds, contradicting the conventional view that only *IGHJ5* is functional in porcine antibody repertoires. Among them, RC pigs showed the lowest *IGHJ3* frequency. Furthermore, RC pigs ranked second in antibody repertoire diversity among the five breeds, implying abundant antigen-binding specificity, and exhibited the lowest CDR3 proportion, indicating breed-specific V(D)J recombination preferences. Conclusions: These results clarify the conserved and breed-specific characteristics of RC pig antibody repertoires, establishing a basis for exploring the genetic regulation of V(D)J-mediated antibody repertoire changes under varied immune conditions. This work also provides genomic support for the rational utilization of RC pig genetic resources.

## 1. Introduction

Pigs are not only an important livestock in economy, but also a valuable model for investigating antibody (Ab) repertoire ontogeny in immunological research. Their remarkable commercial value in the livestock industry underscores the necessity of exploring their immune traits to enhance disease resistance [1]. The porcine epitheliochorial placenta differs from that of humans in blocking the transfer of immunoglobulins (Igs) and other proteins to the fetus. It makes neonatal piglets a model for analyzing Ab repertoire development free from maternal passive influence [2,3,4]. This makes neonatal piglets an ideal animal model for elucidating key immunological issues such as Ab repertoire formation, V(D)J recombination, and antigen (Ag)-binding specificity [2].

China’s indigenous pig breeds constitute a valuable genetic resource pool. The Rongchang (RC) pig, due to its strong adaptability, good disease resistance, excellent meat quality and stable genetic performance, is a nationally protected pig breed [5,6,7,8]. As another typical Chinese indigenous breed, the Bama (BM) pig also has been successfully applied in porcine genetic and immunology research, due to its stable genetic characteristics and unique immune resource advantages [9,10]. RC pigs hold significant importance in ecological and agricultural fields, but the characteristics of the RC pig’s Ab repertoire remain unclear, leading to critical gaps in our understanding of pig immune diversity. To date, research on pig Ab repertoires has been primarily limited to Yorkshire (white) pigs, Landrace pigs, and their Yorkshire–Landrace crossbreds [3,11,12,13,14,15,16], which fails to comprehensively cover the full picture of Ab repertoire utilization across different pig lines—particularly indigenous breeds with unique genetic backgrounds [17]. This has hindered our comprehensive exploration of the evolution of the porcine immune system and limited the development of the unique immune potential of our indigenous breeds.

Generally, the porcine Ab repertoire comprises two Ig heavy chains (IgH) and two Ig light chains (IgL) [18]. The IgH chain comprises a heavy chain variable region (VH) and a constant region (CH) [19]. The VH region contains three adjacent complementarity-determining regions (CDRs), particularly heavy chain complementarity-determining region 3 (HCDR3). The HCDR3 region significantly influences the specificity and diversity recognition of Ig through highly diverse amino acid compositions and arrangements [20]. At the genetic level, the VH region is encoded by gene segments, including the immunoglobulin heavy chain variable (*IGHV*), diversity (*IGHD*), and joining (*IGHJ*) gene segments. The B lymphocyte maturation process generates a diverse Ab repertoire through complex mechanisms [21,22]. The porcine *IGH* locus consists of 5 *IGHJ*, 4 *IGHD* and 15 *IGHV* gene segments [23,24]. Ab repertoire formation is reliant on the selective employment of Ig gene segments, for which *IGHV* and *IGHJ* genes are essential for determining the specific Ag-binding properties of antibodies [21,22,23,24]. Previous studies have shown that only the *IGHJ5* gene in the pig Ab repertoire is functional, while other *IGHJ* family members are considered non-functional or undetectable [3,11,12,13,14,15,16]. However, the universality of *IGHJ* genes across different pig breeds (including native breeds such as RC pigs) has not been verified, which provides insights for analyzing the similarities and differences in Ab repertoires among different pig breeds. The structural complexity of Ig genes further emphasizes the need to validate previous findings across different breeds to avoid overgeneralization.

The lack of research on RC pig Ab libraries not only hinders a comprehensive understanding of the pig immune system’s evolution but also limits the effective utilization of its genetic resources. With China’s increasing emphasis on the protection and utilization of indigenous livestock and poultry breeds [25], elucidating the composition of RC pig Ab libraries and their gene usage patterns has become crucial. This study employed immunomics high-throughput sequencing technology to characterize the Ab repertoire composition of five healthy purebred pigs (native breeds RC and BM and commercial breeds Yorkshire, Duroc, Landrace). By comparing the Ab repertoire differences between RC pigs and the other four breeds, it reveals breed-specific immune characteristics and elucidates adaptive evolution of Ab repertoires under different rearing environments. This work lays the foundation for exploring the regulatory mechanisms of V(D)J (variable, diversity, and joining) gene rearrangement and Ab repertoire specificity changes in RC pigs under various immune states, while providing scientific evidence for the effective utilization of RC pig genetic resources.

## 2. Results

### 2.1. Establishment of High Throughput Sequencing Platform for Analyzing the Porcine Ab Repertoire in Different Breeds

**Figure 1:**
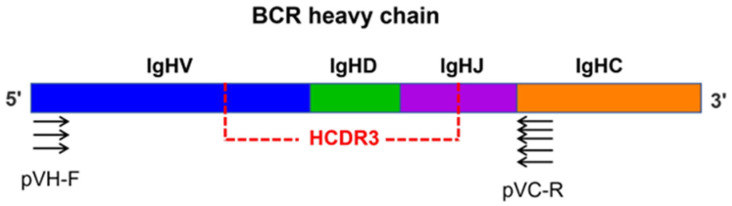
Experimental design. Workflow for amplifying and sequencing the porcine BCR heavy chain via multiplex PCR. Two primer mixtures (three forward primers targeting *IGHV* genes and five reverse primers targeting *IGHC* genes) to amplify full-length variable region of the BCR heavy chain (Table S7). The red dashed line highlights the heavy chain complementarity-determining region 3 (HCDR3), which is encoded by the junction of *IGHV*, *IGHD*, and *IGHJ* gene segments and is critical for antibody diversity and specificity.

Five representative purebred porcine breeds were chosen as experimental subjects, including local porcine breeds RC and BM and the common commercial breeds Landrace pig, Duroc pig and Yorkshire pig. Multiple PCR primers were designed, targeting their BCR heavy chains, and the Ab repertoire composition of these five representative pig breeds was analyzed through immunomics high-throughput sequencing (Figure 1 and Table 1). This study comparatively analyzed three core aspects of data quality, Ab library enrichment efficiency, and Ab library diversity between RC pigs and four other pig breeds. The results demonstrated that the filter efficiency of all pig breeds exceeded 93% (values below 90% indicated substandard raw data quality). Among them, the RC pig (95.58%) demonstrated superior performance compared to the indigenous breed BM and commercial pig breeds Duroc and Yorkshire, while lagging behind Landrace (Table 1). This difference may be attributed to genomic specificity among different pig breeds and primer matching efficiency, among which Landrace, the most commonly used commercial pig breed, exhibits more comprehensive genomic annotation and higher primer compatibility [26,27]. These findings indicate high-quality raw sequencing data and robust experimental process stability. Moreover, all pig breeds achieved a 50% target sequence coverage, confirming that the strategy of specifically enriching target regions (VDJ fragments) showed no interspecies bias, with highly comparable data quality (Table 1). However, RC pigs exhibited the lowest CDR3 sequence coverage (72.16%), suggesting potential breed-specific V(D)J recombination preferences (e.g., higher susceptibility to truncation in the CDR3 region). These results collectively verify the credibility of the high-throughput sequencing dataset from this study on Ab libraries across different pig breeds.

### 2.2. Breed-Specific Diversity in IGHV, IGHD, IGHJ Gene Segment Usage

**Figure 2:**
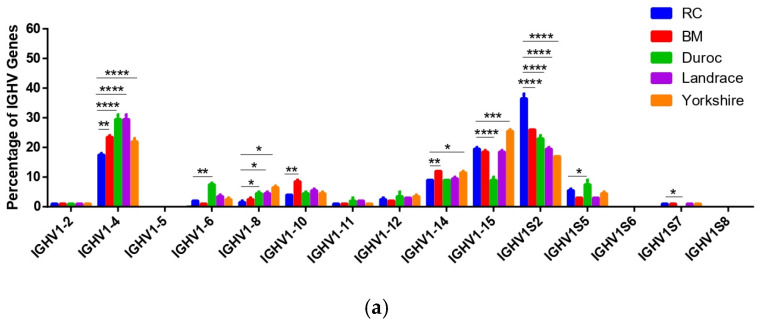

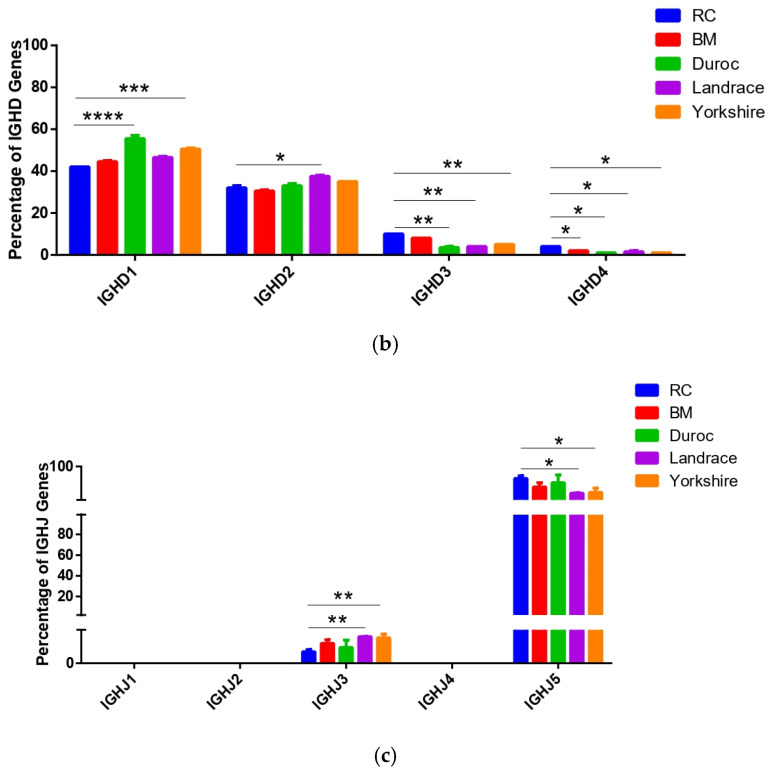
*IGHV*, *IGHD* and *IGHJ* gene segment usage patterns in the *IGH* repertoires of five different porcine breeds. (**a**) The usage proportion of the 15 *IGHV* gene (*Y*–axis) utilized by each of the five porcine breeds (RC, BM, Landrace, Duroc, Yorkshire); (**b**) the usage proportion of 4 *IGHD* genes (*Y*–axis) used by the five porcine breeds (RC, BM, Landrace, Duroc, Yorkshire); and (**c**) the usage proportion of 5 *IGHJ* genes (*Y*–axis) used by the five porcine breeds (RC, BM, Landrace, Duroc, Yorkshire). * *p* ≤ 0.05, ** *p* ≤ 0.01, *** *p* ≤ 0.001, and **** *p* ≤ 0.0001.

Data sourced from the International ImMunoGeneTics information system (IMGT) database indicate that the porcine *IGH* locus consists of 5 *IGHJ*, 4 *IGHD* and 15 *IGHV* gene segments. To investigate the similarities and differences in the distribution of V, D, and J segments among pig breeds, we aligned all high-quality sequences from five pig breeds (RC, BM, Landrace, Duroc, Yorkshire) with reference gene sequences, and further calculated the percentages of *IGHV*, *IGHD* and *IGHJ* segments, relative to the total number of sequencing reads. The results showed that among the 15 *IGHV* genes, all breeds showed a strong preference for four core *IGHV* genes (*IGHV1S2*, *IGHV1-15*, *IGHV1-4*, and *IGHV1-14*), with a combined usage frequency between 69% and 82.5% (Figure 2a and Table S1). Among them, the *IGHV1S2* usage frequency was highest in RC pigs (36.50 ± 0.83%), which was significantly higher than that in BM pigs and the three commercial breeds (*p* < 0.0001) (Figure 2a and Table S1). In contrast, the *IGHV1-4* usage frequency was lowest in RC Pigs (17.50 ± 0.29%), while it was significantly lower than that in BM pigs (*p* < 0.01) and extremely significantly lower than that in the three commercial breeds (*p* < 0.0001) (Figure 2a and Table S1). *IgHV1S7*, annotated as nonfunctional pseudogenes in the IGMT database, was also transcribed in all four porcine breeds except Duroc (Figure 2a).

Among the four *IGHD* genes, *IGHD1* and *IGHD2* are the two most frequently used in five breeds of pigs, with their combined usage rate exceeding 75%. The *IGHD1* usage frequency of RC pigs (42.00 ± 0.00%) was significantly lower than that of Duroc and Yorkshire pigs (*p* < 0.001), while no significant difference was observed between RC and Landrace pigs (*p* > 0.05). For low-frequency *IGHD3* and *IGHD4*, RC pigs had the highest usage frequencies of the two genes: *IGHD3* was significantly higher than that in the three commercial breeds (*p* < 0.01), and *IGHD4* was significantly higher than that in BM pigs and the three commercial breeds (*p* < 0.05) (Figure 2b and Table S2).

Butler and Sinkora, as well as Ji, have all demonstrated that only one *IGHJ* (*IGHJ5*) is functional in the five *IGHJ* genes of normal pigs [3,11,24,28]. Surprisingly, among the five *IGHJ* genes, five piglets not only use *IGHJ5* but also *IGHJ3*, and RC pigs had a higher *IGHJ5* usage frequency (99.65 ± 0.05%) than Landrace and Yorkshire pigs (Figure 2c and Table S3). RC pigs had the lowest *IGHJ3* usage frequency (0.35 ± 0.05%), which was significantly lower than Landrace and Yorkshire pigs (Figure 2c and Table S3). All of these results show that different pig breeds selectively use specific *IGH* VDJ genes.

### 2.3. IgH VJ Analysis of Recombination Diversity in Different Breeds

A variety of antibodies is generated via V(D)J recombination during B lymphocyte maturation [3,20]. V and J gene segments contribute the major proportion of the variable region sequence of immunoglobulins, and the VJ combination preference directly reflects the diversity characteristics of the Ab repertoire. Focusing on VJ recombination allows us to efficiently identify breed-specific immune repertoire signatures. Therefore, to analyze the differences in *IGH* recombination diversity among different breeds and compare the varieties between RC pigs and the other four breeds, we analyzed the *IGH* VJ recombination diversity of different porcine breeds and made statistics for the combination of V–J genes in different porcine breeds. The results showed that the proportion of five pig VJ gene combinations was different, among which RC used more (*IGHV1S2*)–*IGHJ5*, (*IGHV1-15*)–*IGHJ5* and (*IGHV1-4*)–*IGHJ5* in order, accounting for 32.27%, 16.21% and 13.83%, respectively (Table 2). Notably, five porcine breeds also used the *IGHV–IGHJ3* combination, although the proportion of the combination was different, but all were less than 0.3%, among which RC used the (*IGHV1-4*)–*IGHJ3*, (*IGHV1S2*)–*IGHJ3*, (*IGHV1-15*)–*IGHJ3*, (*IGHV1S5*)–*IGHJ3* and (*IGHV1-14*)–*IGHJ3* combination (Table 3). This contradicts previous reports by Butler and Sinkora [24,28] that *IGHJ* only uses *IGHJ5* during *IGH* V(D)J rearrangement. The above results suggest that *IGHJ3* can also be utilized during VDJ rearrangement in different pig breeds, but due to the extensive expression of the *IGHV* gene, VJ recombination varies among different porcine breeds. V(D)J recombination diversity is the molecular basis for generating highly diverse Ab libraries, enabling pigs to recognize, bind, and eliminate various pathogens, thereby enhancing their immune defense capabilities and environmental adaptability [4,28]. The above results indicate that the extensive expression of *IGHV* genes facilitates the formation of V(D)J recombination diversity in different pig breeds, providing an important immunogenetic foundation for the strong adaptability of China local pig breeds (such as RC pigs) [4,28].

### 2.4. IGH CDR3 Amino Acids Length Distribution in Different Breeds

The variable portion of the heavy chain BCR controls the diversity of the Ab repertoire, and the HCDR3 directly interacts with the Ag and establishes the Ag-binding specificity of BCR. Therefore, the diversity of Ab repertoire is mainly determined by HCDR3. The sequencing data showed that there were differences in the overall number of HCDR3 sequences and HCDR3 sequence ratio of five breed healthy piglets (Table 1 and Table S4), and found that the HCDR3 amino acid sequence similarity was low among different individuals, indicating that the body Ab library had rich diversity. Among them, the Ab repertoire capacity of RC (1,294,939) ranks second only to that of Landrace. A larger Ab repertoire capacity indicates richer potential Ag-binding specificity in its Ab pool, making it a high-quality candidate for screening native pig-derived antibodies, which demonstrates the high diversity potential of RC antibodies.

**Figure 3:**
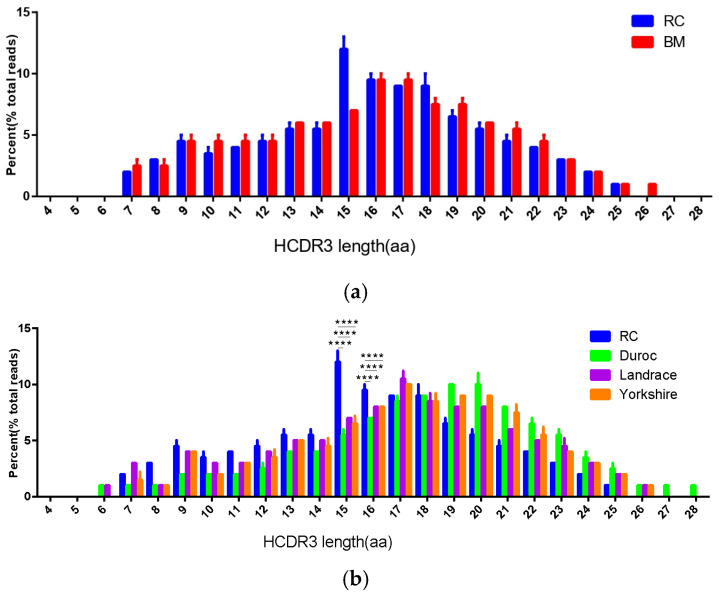

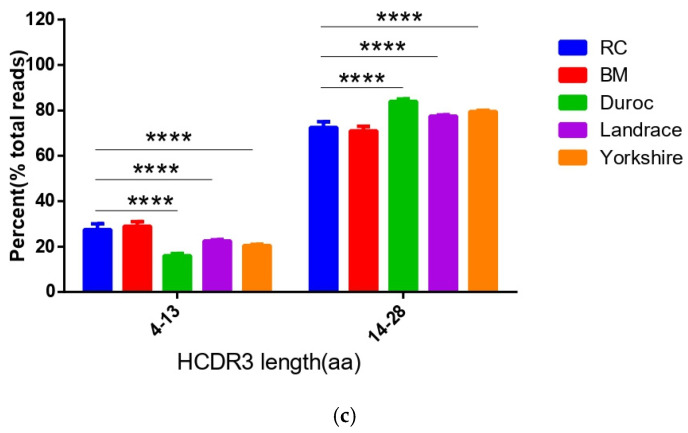
The length profile of HCDR3 length. (**a**) The length profile of HCDR3 covering 4–28 aa was analyzed for RC and BM; (**b**) the length profile of HCDR3 covering 4–28 aa was analyzed for Landrace, Duroc and Yorkshire; and (**c**) analyzing the five pig breeds’ proportions of HCDR3 4–13 aa length and HCDR3 14–28 aa length. **** *p* ≤ 0.0001.

CDR3 length distribution represents an essential indicator for evaluating the diversity characteristics of Ab repertoires [3,29]. We calculated the proportion of HCDR3 sequences with lengths ranging from 4 to 28 amino acids (aa) among different porcine breeds. The Shapiro–Wilk test was used to confirm that the HCDR3 length distribution was normal (Table S5). Results showed that RC and BM showed a similar Gaussian distribution of HCDR3 length, with an average of about 15.00 aa (Figure 3a and Table S5). Landrace, Duroc and Yorkshire pigs also showed a typical Gaussian distribution, with a longer average length of about 16.00 aa (Figure 3b and Table S5). This may be because RC and BM are more inclined to produce antibodies with a shorter CDR3 region or special structure.

Comparing the HCDR3 length distribution among the five porcine breeds, RC and BM show similarities, while RC was significantly different from Landrace, Duroc and Yorkshire (one-way ANOVA, F = 58.72, df = 4/10, *p* < 0.0001) (Figure 3c). In RC and BM, the HCDR3 region with 4–13 amino acids accounts for 26.80–28.10% of the total length, while the HCDR3 region with 14–28 amino acids accounts for 71.90–73.20%. In contrast, in Landrace, Duroc, and Yorkshire, the HCDR3 region with 4–13 amino acids accounts for 15.83–22.63%, and the HCDR3 region with 14–28 amino acids accounts for 77.37–84.17% (Figure 3c and Table S6). Tukey’s multiple comparison test further confirmed that the proportion of 4–13 aa and 14–28 aa HCDR3 sequences in RC pigs were significantly different from those in the three commercial pig breeds (*p* < 0.0001), while no significant difference was found between RC and BM pigs (*p* > 0.05). These results indicated that commercial pig breeds possess a greater potential for HCDR3 length diversity, which may contribute to a broader spectrum of antigen-binding specificity in their Ab repertoires.

## 3. Discussion

In contrast to humans and mice, the porcine *IGH* locus contains a low number of V, D and J germline gene segments, with pigs harboring only 15 *IGHV* genes in total [3,23,24,30,31]. In the report by Butler, Ji and Li et al. [3,11,23,24,28], the utilization of *IGHV* genes within the pre-immune Ab repertoire of healthy pigs is markedly restricted. *IGHV1S2*, *IGHV1-15*, *IGHV1-4* and *IGHV1-14* were predominantly expressed segments in both genomic DNA and the transcriptional products of *IGHV* genes in piglets. In our present study, all 13 identified functional *IGHV* genes were detected in samples, highlighting the sequencing depth of immunomics high-throughput sequencing. Meanwhile, all five pig breeds also preferred to use *IGHV1S2*, *IGHV1-15*, *IGHV1-4* and *IGHV1-14*. In terms of *IGHV1S2* (most frequently used in RC pigs) and *IGHV1-4* (least frequently used in RC pigs), the RC pig showed significant differences compared to the other four pig breeds. These results suggest that the functional genes of *IGHV* are relatively conserved among different pig strains, but their preferences are different. So, different breeds use Ab libraries to respond to different immune responses in a variety of ways. The *IGHV* gene directly determines the structure and recognition scope of antibodies. Different Ab structures result in variations in the types of pathogens recognized, the speed of immune responses, and the intensity of defense. Additionally, influenced by long-term geographical environments, disease pressures, and breed selection, different pig breeds have developed diverse Ab repertoire utilization strategies, ultimately manifesting as differences in immune response patterns.

*IGHD1* and *IGHD2* account for over 75% of all pig breeds, indicating that these two genes are conserved core functional genes that are responsible for Ab basic rearrangement, which are crucial for maintaining fundamental immune responses [4,28]. In contrast, the three commercial pig breeds, which have long been selected for rapid growth and uniform disease resistance, rely more on efficient and broad-spectrum *IGHD1*, hence their higher usage frequency [11,23]. The most critical RC pig breeds, as local varieties, have long adapted to indigenous pathogens and environmental pressures, thus utilizing low-frequency *IGHD3* and *IGHD4* more frequently to enhance Ab diversity in response to specific local pathogens, ultimately forming *IGHD* usage preferences that are distinct from commercial breeds [4].

Previous studies by Li et al. [3,11,24,28] have all demonstrated that the Landrace pigs only have one functional *IGHJ* (*IGHJ5*) gene. However, our results indicate that among these five piglets, the utilization rate of *IGHJ5* exceeded 99.0%, while all five piglets use *IGHJ3*, with its utilization rate ranging between 0.2% and 0.8%. This challenges the traditional view that only functional *IGHJ5* is utilized in the porcine Ab repertoire. Because of the high diversity of antibodies, conventional high-throughput studies failed to detect the low-frequency signal of *IGHJ3* due to limitations in sequencing depth, primer bias, or sample age. With the advancement of sequencing technologies, the information obtained through high-throughput sequencing is invaluable for understanding the Ab characteristics of different species. High-throughput sequencing has been widely used to characterize Ab characteristics in different species such as mouse and human [32,33,34,35], but there are still few studies on the Ab libraries of normal pigs and pigs infected with viruses [3,11,24,28], and most of them are concentrated in purebred Landrace pigs or hybrid breeds of Yorkshire, Duroc and Landrace pigs [3,11,12,13,14,15,16]. In this study, ultra-high-depth sequencing and unbiased experimental design precisely detected the “weak functional utilization” of *IGHJ3*. Among the five pig breeds, the *IGHJ3* utilization frequency in RC pigs was only 0.4%: the lowest level among all pig breeds. The utilization regulation mechanism of the low-frequency expressed *IGHJ3* gene in the Ab repertoire formation process of different pig breeds has inherent differences, which ultimately leads to the RC pigs exhibiting a more significant degree of weak functional utilization of *IGHJ3*, manifested as a utilization frequency far lower than that of the Yorkshire and Landrace pigs. However, further experiments (including single knockout of *IGHJ5* gene, single knockout of *IGHJ3* gene, and simultaneous knockout of *IGHJ5* and *IGHJ3* gene in wild-type RC pigs) are still required to elucidate the regulatory role of *IGHJ5* preference in the Ab repertoire diversity of RC pigs, as well as whether the low-frequency expression of *IGHJ3* affects the overall expression of the *IGH* Ab repertoire.

A variety of antibodies is generated via V(D)J recombination during B lymphocyte maturation [3,15,20,36]. The preference of *IGH* VJ gene combinations is a key characteristic reflecting the diversity of recombinant Ab libraries in pigs, which can effectively identify species-specific immune repertoire features. This study aims to analyze the differences in *IGH* rearrangement diversity among different pig breeds, thus focusing on VJ recombination as an effective method for identifying species-specific immune repertoire features. This study revealed that the VJ combination of RC pigs differs from other pig breeds, reflecting the breed-specific diversity of Ab repertoire recombination—RC pigs exhibit unique VJ recombination preferences. Moreover, all breeds adopt the *IGHV*–*IGHJ3* combination, which contradicts the conclusions previously reported by Butler and Sinkora [3,11,24,28]. Additionally, the *IGHV*–*IGHJ3* combination used by RC pigs has a distinct gene pairing type (*IGHV1-4*/*IGHV1S2*/*IGHV1-15*/*IGHV1S5*/*IGHV1-14* paired with *IGHJ3*), combined with the high-frequency preference for IGHJ5-related combinations, further confirming that RC pigs have developed a unique immune repertoire recombination pattern. The differential VJ repertoire in RC pigs suggests that they may exhibit fundamental differences from other pig breeds in terms of immune response mechanisms, resistance to specific pathogens, and immune adaptability. This provides critical experimental evidence for further exploration of RC pigs’ disease resistance breeding potential and elucidation of the molecular mechanisms underlying their immune characteristics.

CDR3 is the core region of Ab–Ag binding, the total number of its sequences reflects the size of the Ab library, and the sequence ratio reflects the integrity of the CDR3 region in the target sequence. Together, these two factors determine the diversity potential of the Ab library. As an indigenous pig breed, the Ab repertoire of RC pigs (1,294,939) ranks second only to that of Landrace pigs, significantly outperforming Yorkshire, Duroc and BM pigs. This indicates that its Ab repertoire possesses a richer potential Ag-binding specificity, making it suitable for research on the immunogenetic mechanisms of indigenous pig breeds and the preparation of porcine-derived monoclonal antibodies. RC pigs exhibited the lowest proportion of CDR3 sequences (72.16%), suggesting a species-specific V(D)J recombination preference. This implies that RC pigs may be more inclined to generate antibodies with shorter or structurally unique CDR3 regions, which could serve as a breakthrough for subsequent identification of species-specific Ab genes.

CDR3 length distribution represents an essential indicator for evaluating the diversity characteristics of Ab repertoires [3,29]. This study compared the HCDR3 length distribution across five pig breeds and found that RC and BM exhibited similarities, while showing significant differences from Landrace, Duroc, and Yorkshire pigs. This suggests that RC and BM are more prone to producing antibodies with shorter or structurally unique CDR3 regions, whereas the HCDR3 length distribution characteristics of commercial pig breeds reflect their greater potential for genetic diversity in Ab libraries.

In conclusion, we systematically analyzed the composition of Ab repertoire from five representative pig breeds, including the RC pig, by immunomics high-throughput sequencing. We elucidated that the usage of V, D, and J genes in *IGH* of different pig Ab libraries exhibits high selectivity and differences in their compositional features. In summary, these results not only fill the gap in the Ab repertoire of Chinese indigenous pig breeds (RC pigs), enrich the pig immune database, and lay the foundation for exploring the regulatory mechanisms of specific changes in the V(D)J rearranged Ab repertoire of RC pigs under different immune states, but also lay a molecular foundation for breeding for disease resistance and the utilization of genetic resources in RC pigs, further contributing to the development of pig breeds with fewer diseases and stronger resistance, and promoting the effective utilization of RC pig genetic resources.

## 4. Materials and Methods

### 4.1. Animals

All procedures adhered to the Animal Ethics Committee of Chongqing Academy of Animal Sciences, China (XKY-202407B01) guidelines.

Five healthy purebred porcine breeds (RC, BM, American Landrace, American Duroc and American Yorkshire) were collected from Shuanghe Pig Farm, Chongqing Academy of Animal Sciences. Three piglets of each breed were selected from the same litter: a total of 15 piglets from five different breeds. All piglets had unified feeding and management conditions and were without any specific vaccine immunization or pathogen infection. All experiments and procedures were approved by the Animal Experiment Ethics Committee of Chongqing Academy of Animal Sciences.

### 4.2. Porcine Peripheral Blood Mononuclear Cell (PBMC) Isolation

Blood samples were collected from the five porcine breeds for PBMC isolation at 3 weeks old. The isolation kit for PBMCs was consistent with that used by Ji et al. [3], employing the Pig Peripheral Blood Mononuclear Cell Isolation Kit (TBD Science, Tianjin, China). PBMCs were strictly isolated from fresh pig whole blood. The specific steps were as follows: 10 mL heparin sodium anticoagulated whole blood was diluted with 5 mL phosphate-buffered saline (PBS). Slowly add the diluted pig blood sample to a sterile centrifuge tube containing 15 mL of lymphocyte isolation buffer to prevent sample mixing. Then, centrifuge at 500× *g* for 30 min at room temperature to form a distinct PBMC-enriched layer. Carefully aspirate the enriched layer at the sample-medium interface with a sterile pipette and transfer it to a new 15 mL sterile centrifuge tube. The collected PBMCs were washed twice with 10 mL of washing solution, and after each wash, centrifuge at 250× *g* for 10 min to precipitate the cells and remove residual medium contaminants. Finally, discard the supernatant after centrifugation, and immediately use the purified PBMCs for total RNA extraction.

### 4.3. Library Preparation and Immunomics High-Throughput Sequencing

GENEWIZ Inc. (Suzhou, China) carried out the deep sequencing of the Ig repertoire. RNA extraction, cDNA synthesis, and PCR amplification were all part of the library preparation process. Following the manufacturer’s operational procedures, RNA was extracted from 1 × 10^6^ PBMCs using the Qiagen RNeasy Mini Kit (Qiagen, Hilden, Germany), and a Qubit Fluorometer (Thermo Fisher Scientific, Waltham, MA, USA) was used to measure the amount of RNA. Then, cDNA was created using the PrimeScript RT Reagent Kit (Perfect Real Time) (Takara, Kusatsu, Shiga, Japan). Multiplex PCR was used to amplify *IGH* libraries. Two primer mixtures (three forward primers targeting *IGHV* genes and five reverse primers targeting *IGHC* genes) (Table S7) were used to amplify the full-length variable region of the BCR heavy chain, using Phanta Max Super-Fidelity DNA Polymerase (Vazyme, Nanjing, China). A total of 40 pmol of two primer mixtures and 2 μL cDNA were added to the reaction for each sample, bringing the total volume to 50 μL. A step of 95 °C for 3 min was the first in the PCR protocol. This was followed by a phase that included 33 cycles of 95 °C for 15 s, 62 °C for 15 s, and 72 °C for 1 min. After cycling, there was a final chilling phase at 4 °C and a 5 min elongation stage at 72 °C. As previously mentioned, libraries were sequenced using the Illumina MiSeq 2 × 300 platform after quality control on a Bioanalyzer High Sensitivity DNA chip (Agilent, Santa Clara, CA, USA).

### 4.4. Sequence Analysis

The analysis of the original Illumina PE250 data was performed following the bioinformatics workflow developed by Genewiz, as described by Butler, J.E [2]. In a nutshell, using conventional Illumina criteria, MiSeq reads Fastq data were QC filtered. Along with terminal nucleotides that had Q scores of less than 20, sequencing primers and adaptors were cut. Using Pandaseq, the quality-filtered paired readings were put together. The clean reads were then mapped to the V, D, and J genes, and CDR3s were extracted using the IgBLAST program (http://www.ncbi.nlm.nih.gov/igblast, accessed on 7 January 2025) and the IMGT database (IMGT, http://www.imgt.org, accessed on 7 January 2025).

### 4.5. Statistical Analysis

Statistical analyses were performed with the pig breed as the sole independent variable, including the frequency of *IGHV*/*IGHD*/*IGHJ* genes, Gaussian distribution validation, and length distribution analysis of HCDR3 (amino acid length frequency and proportions of 4–13 and 14–28 aa subgroups). All analyses were based on three biological replicates and carried out using GraphPad Prism 9.4.1 (GraphPad Software, San Diego, CA, USA). Results are expressed as mean ± standard error (SE). Significance levels were set as follows: * *p* ≤ 0.05, ** *p* ≤ 0.01, *** *p* ≤ 0.001, and **** *p* ≤ 0.0001, while *p* > 0.05 was regarded as representing no statistically significant difference. The Shapiro–Wilk test was used to evaluate the normality of residuals (the essential prerequisite for ANOVA), and Levene’s test was used to validate the homogeneity of variances of raw data. Normally distributed residuals and variance-homogeneous data were analyzed by one-way ANOVA, followed by Tukey’s multiple comparisons. Non-normally distributed data were examined using the Kruskal–Wallis H test with Dunn’s multiple comparisons and Benjamini–Hochberg correction. The Shapiro–Wilk test was also applied to validate the Gaussian distribution of full-length HCDR3 (4–28 aa) as a descriptive characteristic of the data itself. HCDR3 length distribution was analyzed using the same statistical procedure as gene frequency analysis.

## Figures and Tables

**Table 1:** Summary of sequencing comparison.

Sequence Comparison	RC (%)	BM (%)	Duroc (%)	Landrace (%)	Yorkshire (%)
Filter efficiency (Filtered/Total)	95.58	93.02	93.21	96.13	93.79
Target sequence ratio (Target/Filtered)	50.00	50.00	50.00	50.00	50.00
CDR3 sequence ratio (CDR3/Target)	72.16	72.95	75.19	78.06	75.42

**Table 2:** The frequency of the five porcine breed *IGHV–IGHJ5* gene usage.

*IGHV*–*IGHJ5* Combination	RC	BM	Duroc	Landrace	Yorkshire
(*IGHV1-2*)–*IGHJ5*	0.51	0.70	0.41	0.65	0.44
(*IGHV1-4*)–*IGHJ5*	13.83	21.22	29.00	27.35	21.90
(*IGHV1-6*)–*IGHJ5*	1.62	0.97	5.79	2.62	2.23
(*IGHV1-8*)–*IGHJ5*	1.60	2.40	3.84	4.04	6.50
(*IGHV1-10*)–*IGHJ5*	3.16	7.50	3.20	5.15	4.79
(*IGHV1-11*)–*IGHJ5*	0.36	1.21	1.12	1.36	1.20
(*IGHV1-12*)–*IGHJ5*	1.47	1.82	2.11	2.26	3.15
(*IGHV1-14*)–*IGHJ5*	7.24	10.36	8.15	8.06	9.91
(*IGHV1-15*)–*IGHJ5*	16.21	16.58	6.58	16.59	24.01
(*IGHV1S2*)–*IGHJ5*	32.27	23.12	22.00	16.25	15.85
(*IGHV1S5*)–*IGHJ5*	5.43	2.73	8.51	2.74	3.37
(*IGHV1S7*)–*IGHJ5*	0.72	0.63	0.34	0.48	0.68

**Table 3:** The frequency of the five porcine breed *IGHV*–*IGHJ3* gene usage.

*IGHV*–*IGHJ3* Combination	RC	BM	Duroc	Landrace	Yorkshire
(*IGHV1-2*)–*IGHJ3*	0	0.03	0	0	0
(*IGHV1-4*)–*IGHJ3*	0.11	0.10	0.08	0.26	0.13
(*IGHV1-6*)–*IGHJ3*	0	0	0	0	0.01
(*IGHV1-8*)–*IGHJ3*	0	0	0.01	0.02	0
(*IGHV1-10*)–*IGHJ3*	0	0.13	0	0	0.07
(*IGHV1-11*)–*IGHJ3*	0	0	0	0	0
(*IGHV1-12*)–*IGHJ3*	0	0	0	0	0.07
(*IGHV1-14*)–*IGHJ3*	0.02	0.04	0.02	0.03	0.03
(*IGHV1-15*)–*IGHJ3*	0.06	0.09	0.13	0.28	0.33
(*IGHV1S2*)–*IGHJ3*	0.08	0.11	0.03	0.07	0.04
(*IGHV1S5*)–*IGHJ3*	0.03	0	0	0	0.04
(*IGHV1S7*)–*IGHJ3*	0	0	0	0	0

## Data Availability

The raw data supporting the conclusions of this article will be made available by the authors upon request.

## Supplementary Materials

The following supporting information can be downloaded at: https://www.mdpi.com/article/10.3390/genes17030262/s1, Table S1: Analysis of the significant difference in the usage frequency of five pig *IGHV* genes; Table S2: Analysis of the significant difference in the usage frequency of five pig *IGHD* genes; Table S3: Analysis of the significant difference in the usage frequency of five pig *IGHJ* genes; Table S4: Summary of sequencing reads alignment; Table S5: Descriptive normality test of HCDR3 length in five pig breeds (based on raw data); Table S6: Proportions of HCDR3 with 4–13 aa and 14–28 aa lengths in five pig breeds; Table S7: Primers used for multiplex PCR amplification.
