# Supplementary material for: Genomic Profiling of Antibody Repertoire in Rongchang Pigs Uncovers Conserved V(D)J Gene Usage and Breed-Specific Diversification
Source: Genes (Basel). 2026 Feb 26;17(3):262. doi: 10.3390/genes17030262 (PMC13026311; doi:10.3390/genes17030262)
Supplement: Supplementary file 1 [file genes-17-00262-s001.zip › genes-4147462-supplementary.pdf]

Supplementary Materials

Tables S1-S3 have been revised to comply with the prerequisite of ANOVA: the Shapiro-Wilk test was performed on residuals (instead of raw data) to verify normality, ensuring statistical rigor.”

K-W = Kruskal-Wallis H nonparametric test, ANOVA = one-way analysis of variance; post-hoc analysis for ANOVA used Tukey's multiple comparison, post-hoc analysis for K-W used Dunn's multiple comparison + Benjamini-Hochberg correction; N=3, *P≤0.05, **P≤0.01, ***P≤0.001, ****P<0.0001

Table S1. Analysis of the significant difference in the usage frequency of five pig IGHV genes

Gene Name	RC (mean±SE,%)	BM (mean±SE,%)	Duroc (mean±SE,%)	Landrace (mean±SE,%)	Yorkshire (mean±SE,%)	Residuals Shapiro-Wilk test (*P* value)	Levene's test (*P* value)	Main test methods and results (statistics/df/*P* value)
IGHV1S2	36.50±0.83	26.00±0.00	23.00±0.58	19.50±0.29	17.00±0.00	0.825	0.23	ANOVA/F=126.58/4,10/<0.001
IGHV1-4	17.50±0.29	23.50±0.29	28.00±0.58	29.00±0.58	22.00±0.29	0.793	0.17	ANOVA/F=68.35/4,10/<0.001
IGHV1-14	9.00±0.00	12.00±0.00	9.00±0.00	9.50±0.29	11.50±0.29	0.689	0.28	ANOVA/F=15.63/4,10/0.001
IGHV1-15	19.50±0.29	18.50±0.29	9.00±0.58	18.50±0.00	25.50±0.29	0.712	0.31	ANOVA/F=39.82/4,10/<0.001
IGHV1S5	5.50±0.29	3.00±0.00	7.50±0.87	3.00±0.00	4.50±0.29	0.032	0.15	K-W/χ^2=9.87/4/0.043
IGHV1-10	4.00±0.00	8.50±0.29	4.50±0.29	5.50±0.29	4.50±0.00	0.805	0.24	ANOVA/F=18.72/4,10/0.001
IGHV1-12	2.50±0.29	2.00±0.00	2.50±0.58	3.00±0.00	3.50±0.29	0.766	0.35	ANOVA/F=3.21/4,10/0.058
IGHV1-6	2.00±0.00	1.00±0.00	7.50±0.29	3.50±0.29	2.50±0.29	0.028	0.21	K-W/χ^2=11.82/4/0.019
IGHV1-8	1.50±0.29	2.50±0.29	4.50±0.00	4.50±0.00	6.50±0.29	0.019	0.18	K-W/χ^2=13.05/4/0.011
IGHV1-2	1.00±0.00	1.00±0.00	0.00±0.00	1.00±0.00	1.00±0.00	0.045	0.98	K-W/χ^2=12.35/4/0.016
IGHV1-11	1.00±0.00	1.00±0.00	2.00±0.58	2.00±0.00	1.00±0.00	0.049	0.91	K-W/χ^2=8.96/4/0.062

IGHV1S7	1.00±0.00	1.00±0.00	0.00±0.00	1.00±0.00	1.00±0.00	0.045	0.98	K-W/ χ^2=12.35/4/0.016

Table S2. Analysis of the significant difference in the usage frequency of five pig IGHD genes

Gene Name	RC (mean±SE,%)	BM (mean±SE,%)	Duroc (mean±SE,%)	Landrace (mean±SE,%)	Yorkshire (mean±SE,%)	Residuals Shapiro-Wilk test (P value)	Levene's test (*P* value)	Main test methods and results (statistics/df/P value)
IGHD1	42.00±0.00	44.50±0.29	55.50±0.87	46.50±0.29	50.50±0.29	0.837	0.19	ANOVA/F=28.76/4,10/<0.001

IGHD2	32.00±0.58	30.50±0.29	33.00±0.58	37.50±0.29	35.00±0.00	0.781	0.42	ANOVA/F=4.32/4,10/0.023
IGHD3	10.00±0.00	8.00±0.00	3.50±0.29	4.00±0.00	5.00±0.00	0.036	0.27	K-W/χ^2=14.89/4/0.005
IGHD4	4.00±0.00	2.00±0.00	1.00±0.00	1.50±0.29	1.00±0.00	0.025	0.33	K-W/χ^2=13.27/4/0.010

Table S3. Analysis of the significant difference in the usage frequency of five pig IGHJ genes

**Gene Name**	**RC (mean±SE,%)**	**BM (mean±SE,%)**	**Duroc (mean±SE,%)**	**Landrace (mean±SE,%)**	**Yorkshire (mean±SE,%)**	**Residuals Shapiro-Wilk test (P value)**	**Levene's test (*P* value)**	**Main test methods and results (statistics/df/*P* value)**
IGHJ5	99.65±0.05	99.40±0.05	99.52±0.09	99.20±0.00	99.24±0.05	0.774	0.37	ANOVA/F=3.89/4,10/0.031
IGHJ3	0.35±0.05	0.60±0.05	0.48±0.09	0.80±0.00	0.76±0.05	0.018	0.29	K-W/χ^2=13.89/4/0.007

Table S4. Summary of sequencing reads alignment

**Sequence**	**RC**	**BM**	**Duroc**	**Landrace**	**Yorkshire**
Total sequence reads	3755334	2576236	2835734	3771676	3156426
Filtered sequence reads	3589196	2396520	2643226	3625588	2960346
Target sequence number	1794598	1198260	1321613	1812794	1480173
Total CDR3 sequence number	1294939	874026	993867	1415084	1116362

Table S5. Descriptive normality test of HCDR3 length in five pig breeds (based on raw data)

Pig breed	Shapiro-Wilk test (W value)	Shapiro-Wilk test (*P* value)	Normality test (Gaussian distribution)	Remarks
RC	0.986	0.952	normal	Peak value: 15aa, mean value: ~15.00aa
BM	0.978	0.915	normal	Peak value: 15aa, mean value: ~15.00aa
Duroc	0.981	0.930	normal	Peak value: 16aa, mean value: ~16.00aa
Landrace	0.975	0.898	normal	Peak value: 16aa, mean value: ~16.00aa
Yorkshire	0.983	0.936	normal	Peak value: 16aa, mean value: ~16.00aa

This table presents the descriptive normality test of HCDR3 length itself (raw data), reflecting the inherent distribution characteristics of the data.

Table S6. Proportions of HCDR3 with 4–13 aa and 14–28 aa lengths in five pig breeds

Gene Name	RC (mean±SE,%)	BM (mean±SE,%)	Duroc (mean±SE,%)	Landrace (mean±SE,%)	Yorkshire (mean±SE,%)	Residuals Shapiro-Wilk test (P value)	Levene's test (*P* value)	Main test methods and results (statistics/df/*P* value)
4-13 aa	26.80±1.17	28.10±0.64	15.83±0.44	22.63±0.29	20.17±0.34	0.785	0.305	ANOVA/F=58.72/4,10/<0.0001
14-28 aa	73.20±1.17	71.90±0.64	84.17±0.44	77.37±0.29	79.83±0.34	0.812	0.302	ANOVA/F=58.89/4,10/<0.0001

Table S7. Primers used for multiplex PCR amplification.

Primer Name	Primer Sequence (5′→3′)
pVH-F1	GAGGTGAAGCTGGTGGAGTGTG

pVH-F2	GAGGAGAAGCTGGTGGAGTCTG
pVH-F3	CAGGAGAAGCTGGTGGAGTCTG
pVC-R1	GGCCAGAGGGTAGACCGAT
pVC-R2	AGTCAGTGGGAAGATTTTG
pVC-R3	GACGAGGGGGAAGAGACTC
pVC-R4	AGTCAAGGGGTAGACCAGT
pVC-R5	GACGAGGGGGAAGAGATTC
